# A tumorigenicity evaluation platform for cell therapies based on brain organoids

**DOI:** 10.1186/s40035-024-00446-5

**Published:** 2024-10-29

**Authors:** Jun Xue, Youjun Chu, Yanwang Huang, Ming Chen, Meng Sun, Zhiqin Fan, Yonghe Wu, Liang Chen

**Affiliations:** 1 Department of Neurosurgery, Huashan Hospital, MOE Frontiers Center for Brain Science, Fudan University, Shanghai, 200040 China; 2 Shanghai Institute for Advanced Immunochemical Studies, ShanghaiTech University, Shanghai, 201210 China; 3 National Center for Neurological Disorders, Shanghai Key Laboratory of Brain Function and Restoration and Neural Regeneration, Huashan Hospital, Fudan University, Shanghai, 200040 China; 4 Shanghai Clinical Research and Trial Center, Shanghai, 201210 China

**Keywords:** Tumorigenicity evaluation, Cell therapies, Brain organoids

## Abstract

**Background:**

Tumorigenicity represents a critical challenge in stem cell-based therapies requiring rigorous monitoring. Conventional approaches for tumorigenicity evaluation are based on animal models and have numerous limitations. Brain organoids, which recapitulate the structural and functional complexity of the human brain, have been widely used in neuroscience research. However, the capacity of brain organoids for tumorigenicity evaluation needs to be further elucidated.

**Methods:**

A cerebral organoid model produced from human pluripotent stem cells (hPSCs) was employed. Meanwhile, to enhance the detection sensitivity for potential tumorigenic cells, we created a glioblastoma-like organoid (GBM organoid) model from *TP*53^*−/−*^*/PTEN*^*−/−*^ hPSCs to provide a tumor microenvironment for injected cells. Midbrain dopamine (mDA) cells from human embryonic stem cells were utilized as a cell therapy product. mDA cells, hPSCs, mDA cells spiked with hPSCs, and immature mDA cells were then injected into the brain organoids and NOD SCID mice. The injected cells within the brain organoids were characterized, and compared with those injected in vivo to evaluate the capability of the brain organoids for tumorigenicity evaluation. Single-cell RNA sequencing was performed to identify the differential gene expression between the cerebral organoids and the GBM organoids.

**Results:**

Both cerebral organoids and GBM organoids supported maturation of the injected mDA cells. The hPSCs and immature mDA cells injected in the GBM organoids showed a significantly higher proliferative capacity than those injected in the cerebral organoids and in NOD SCID mice. Furthermore, the spiked hPSCs were detectable in both the cerebral organoids and the GBM organoids. Notably, the GBM organoids demonstrated a superior capacity to enhance proliferation and pluripotency of spiked hPSCs compared to the cerebral organoids and the mouse model. Kyoto Encyclopedia of Genes and Genomes analysis revealed upregulation of tumor-related metabolic pathways and cytokines in the GBM organoids, suggesting that these factors underlie the high detection sensitivity for tumorigenicity evaluation.

**Conclusions:**

Our findings suggest that brain organoids could represent a novel and effective platform for evaluating the tumorigenic risk in stem cell-based therapies. Notably, the GBM organoids offer a superior platform that could complement or potentially replace traditional animal-based models for tumorigenicity evaluation.

**Supplementary Information:**

The online version contains supplementary material available at 10.1186/s40035-024-00446-5.

## Background

Human pluripotent stem cells (hPSCs) provide unique opportunities for cell therapies due to their unlimited self-renewal and pluripotent differentiation capacities [1, 2]. However, the potential of tumorigenicity remains a substantial challenge to clinical applications of stem cell-based therapies [1, 3, 4]. Therefore, rigorous and reliable tumorigenicity assays are needed to ensure safety of stem cell-derived therapeutic products.

Traditionally, preclinical tumorigenicity evaluation of stem cell-based therapies is performed in immunocompromised rodents [5–12]. Nonetheless, these animal models may not accurately predict tumor formation in humans, as evidenced by several case studies where patients developed tumors following stem cell therapies [13–15]. Significant differences in development, macroscopic architecture, cellular composition, and gene expression between human and rodent brains challenge the validity of these animal models [16, 17]. In addition, ethical and welfare concerns arise from the use of large cohorts of mice, sometimes even exceeding a hundred in preclinical studies [9–11, 18]. Furthermore, the long-term experiment time of xenogeneic models, spanning months to years, can significantly postpone the delivery of therapeutic interventions to patients. Hence, there is an urgent need to develop a novel tumorigenicity evaluation platform that could more closely mirror the human brain and could replace animal models.

Brain organoids are three-dimensional (3D) self-organized neural constructs that recapitulate the human brain [19–22]. They reflect the transcriptomic and epigenomic profiles of the fetal brain and exhibit structural features, including the outer subventricular and radial glial zones. As such, they represent a groundbreaking tool for investigating neural development, human evolution, and psychiatric and neurodegenerative diseases, as well as for drug screening [23–26]. Additionally, recognized by regulatory agencies such as the International Society for Stem Cell Research [5], the European Medicines Agency, and the Center for Drug Evaluation of China, organoids serve as a valuable complement to animal models for efficacy and safety assessments of cell therapies when conventional models are impractical. Furthermore, previous research has shown that human neural stem cells (NSCs) injected into cerebral organoids undergo proper differentiation [27, 28]. Reumann et al. [29] demonstrated survival and maturation of ventral midbrain progenitors following injection into ventral midbrain-striatum-cortex assembloids. These findings underscore the potential utility of brain organoids for assessing cell therapies. However, the capacity to identify potential risk cells in brain organoids and the performance of brain organoids as a novel platform for tumorigenicity evaluation remain to be fully elucidated.

In this study, we aimed to evaluate the capacity and sensitivity of brain organoids in assessing tumorigenicity of stem cell-based therapies, in comparison to traditional in vivo models. Therefore, we employed the cerebral organoids derived from hPSCs, and to enhance the sensitivity for detecting potential risk cells in cell products, we generated glioblastoma-like organoids (GBM organoid) from *TP*53^*−/−*^*/PTEN*^*−/−*^ hPSCs. Midbrain dopamine (mDA) cells derived from human embryonic stem cells (hESCs), which are applied in the treatment of Parkinson’s disease (PD), were used as a representative example of cell therapy products.

## Methods

### hPSC maintenance

The H9 line of hESCs was applied as hPSCs. H9 cells were maintained in Matrigel (Corning, 354277, Corning, NY)-coated dishes using NutriStem hPSC XF (Biological Industries, 05-200-1A, Kibbutz Beit Haemek, Israel) with daily medium change in a 37 °C incubator with 5% CO_2_. Upon reaching 70%–80% confluence, cells were detached using EDTA (Invitrogen, 15575–020, Carlsbad, CA) and passaged onto new Matrigel-coated plates. ROCK (Rho-associated coiled-coil-containing protein kinase) inhibitor Y-27632 (TargetMol, T1725, 10 μΜ, Shanghai, China) was added to the passaged medium to prevent cell apoptosis.

### Generation of mDA cells derived from hESCs

**Fig. 1:**
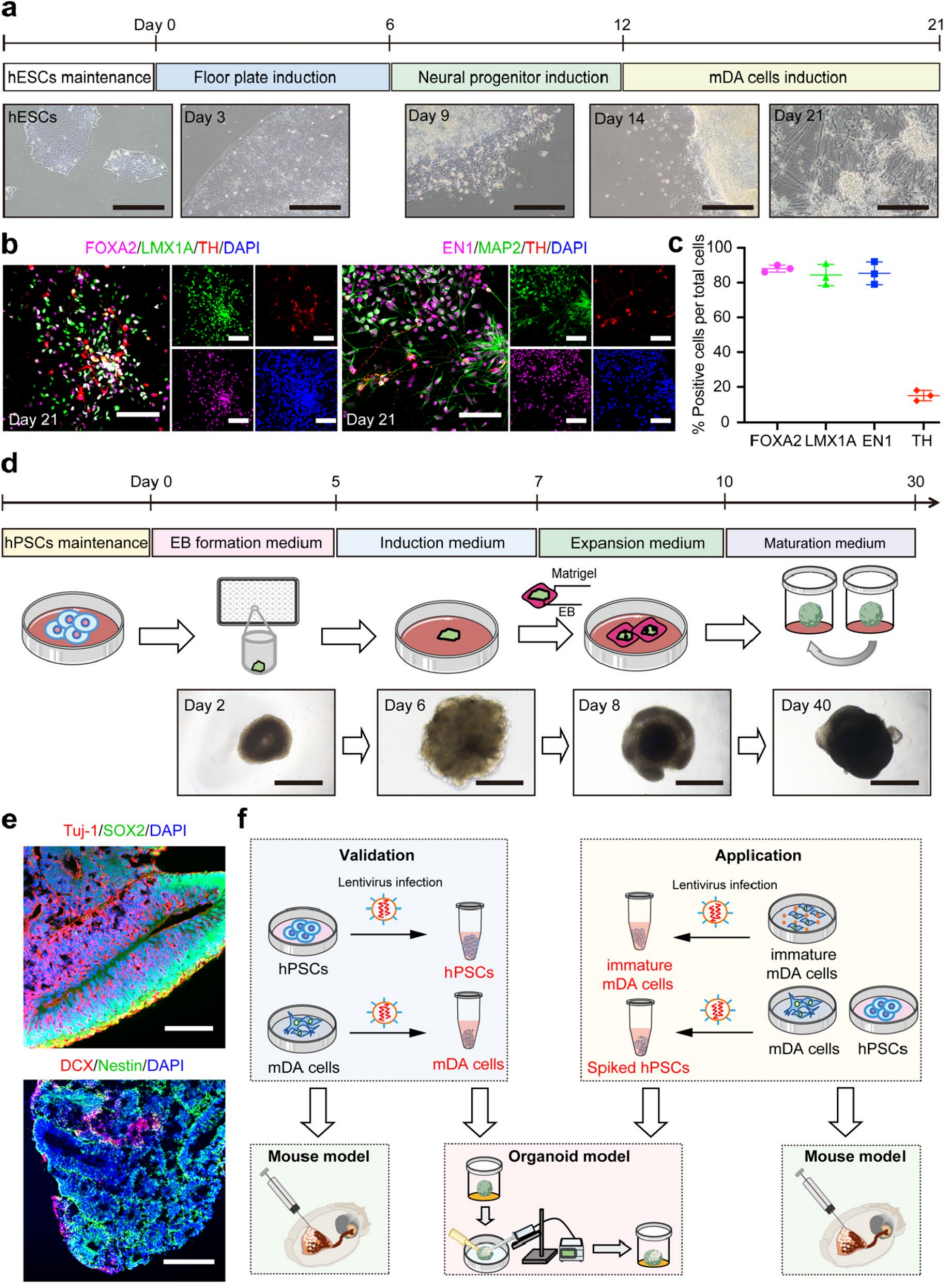
Generation of mDA cells and cerebral organoids. **a** The differentiation protocol and bright-field images of mDA cells derived from hESCs. Scale bars: 400 μm. **b** Immunofluorescence images of mature neurons (MAP2), midbrain dopamine progenitors (FOXA2/LMX1A/EN1), and mature dopamine neurons (TH) at day 21 of mDA cell differentiation. Scale bars: 80 μm. **c** Quantification of FOXA2^+^, LMX1A^+^, EN1^+^ and TH^+^ populations in mDA cells at day 21 of differentiation. Mean ± SD, *n* = 3 batches of differentiation. **d** The generation protocol and bright-field images of cerebral organoids derived from hPSCs. Scale bars: 1000 μm. **e** Immunofluorescence images of neural precursor cells (SOX2/Tuj-1/DCX/Nestin) at day 30 of the cerebral organoids. Scale bars: 200 μm. **f** Schematic of organoid injection and mouse transplantation with hPSCs, mDA cells, immature mDA cells, or mDA cells spiked with hPSCs

H9 cells were differentiated into mDA cells as described previously with minor modifications [30, 31] (Fig. 1a). Briefly, on day 0, H9 colonies were detached with EDTA for 5 min, harvested, and re-seeded in Matrigel-coated spots (~ 1000 cells per spot) with NutriStem medium supplemented with Y-27632. On days 1–6, cells were cultivated in DMEM (Gibco, 11965118, Grand Island, NY) containing 15% Knockout Serum Replacement (KSR, Gibco, 10828028) and *L*-Glutamine (*L*-Glu, Gibco, 25030081). On days 6–12, the cells were incubated with DMEM containing KSR (11.5% days 6–8, 7.5% days 8–10, 3.75% days 10–12), N2 supplement (Gibco, 17502048, 0.25% days 6–8, 0.5% days 8–10, 0.75% days 10–12), *L*-Glu and non-essential amino acid (Gibco, 11140050). On days 12–21, the culture medium was replaced by DMEM/F12 (Gibco, 11320033) with 1% N2 supplement. Additionally, specific chemicals and growth factors were added at various stages: β-mercaptoethanoer (Gibco, 21985023) and SB431542 (MedChemExpress, HY-10431, 10 μΜ, Shanghai, China) added on days 1–8, and LDN193189 (TargetMol, T6158, 0.2 μΜ) supplemented on days 1–12. Sonic hedgehog (PeproTech, 100–45, 100 ng/ml, Rocky Hill, NJ), FGF8 (PeproTech, 100–25, 100 ng/ml), and purmorphamine (MedChemExpress, HY-15108, 2 μΜ) were supplemented on days 2–10. CHIR99021 (TargetMol, T2310, 1 μΜ) was supplemented on days 4–14. On day 9, cells were treated with 40 μM Quercetin (Sigma, Q4951, St. Louis, MO) for 16 h. On days 12–21, ascorbic acid (Sigma, A4403, 200 μΜ), brain-derived neurotrophic factor (PeproTech, 450–02, 20 ng/ml), glia-derived neurotrophic factor (PeproTech, 450–10, 20 ng/ml), dibutyryl cyclic adenosine monophosphate (Sigma, D0627, 500 μΜ), TGF-β3 (PeproTech, 100-36E, 1 ng/ml), and DAPT (TargetMol, T6202, 10 μΜ) were included. On day 15 of differentiation, cells were dissociated with accutase (Sigma, SCR005) and replated onto poly-*L*-ornithine (Sigma, P4957)/Fibronectin (Sigma, F0895)/Laminin (Sigma, L2020)-coated dishes.

### Generation of cerebral organoids and GBM organoids

Cerebral organoids and GBM organoids were generated from hPSCs and *TP*53^*−/−*^*/PTEN*^*−/−*^ hPSCs, respectively, as previously reported [32], using the STEMdiff^™^ cerebral organoid kit (Stemcell Technologies, 08570, Vancouver, Canada) and the STEMdiff^™^ cerebral organoid maturation kit (Stemcell Technologies, 08571). On day 0, hPSCs or *TP*53^*−/−*^*/PTEN*^*−/−*^ hPSCs were dissociated into single cells and reseeded in the 96-well ultra-low attachment plates with embryoid body (EB) formation medium supplemented with Y-27632. On days 2–4, the culture medium was replaced by the EB formation medium without Y-27632. On days 5–6, 1–2 EBs were carefully transferred to the 24-well ultra-low attachment plate with EB induction medium. On day 7, EBs were embedded in Matrigel and cultured in EB expansion medium for three days. On day 10, the medium was replaced with maturation medium. Thereafter, the plate of brain organoids was placed on an orbital shaker and the maturation medium was changed every 2–3 days. The schematic of cerebral organoid generation is shown in Fig. 1d.

### Virus package and labeling of injected cells

HEK293T cells were maintained in DMEM supplemented with 10% FBS. After reaching 70%–90% confluency, cells were co-transfected with three plasmids: the envelope plasmid pMD2.G, the packaging plasmid psPAX2, and the receptor plasmid pHHLVX-EF1α-luciferase/RFP-puro containing either RFP or luciferase, mixed in a 1:1:2 ratio. The transfection mix was prepared by adding the plasmid mix into a tube containing polyethyleneimine in OptiMEM (Gibco, 31985062). The transfection mix was added dropwise to HEK293T cells after incubation at room temperature for 20 min. Virus particles were collected 48 h after transfection and concentrated with Lenti-X^™^ Concentrator (Takara, 631232, Beijing, China). The concentrated virus was aliquoted and stored at − 80 °C for long-term use. hPSCs, mDA cells and immature mDA cells for organoid injection were transfected with the lentivirus. Successful transfection of the RFP signal was confirmed by an immunofluorescence microscope (Olympus, CKX53, Tokyo, Japan), and the luciferase signal was confirmed by bioluminescence imaging (Bio-Real Sciences, Quick View 3000, Austria).

### Organoid injection

Prior to organoid injection, all necessary injection tools, including needles and automatic injection pumps, were disinfected with alcohol and then subjected to ultraviolet disinfection. The fluorescent protein-labeled hPSCs, mDA cells, immature mDA cells, or mDA cells spiked with hPSCs were routinely digested, and their concentrations in the cell suspension were adjusted to be 50,000 cells/μl. The brain organoid was carefully aspirated using a cut pipette tip and placed in a 6-cm dish. Then, 4 μl of the cell suspension was automatically injected by a Hamilton syringe (Hamilton Company) attached to an automatic microinjection pump, into the interior of the brain organoid at a rate of 1 μl/min. Attention was paid to ensure that the cells were not injected into the Matrigel surrounding the brain organoid. During the injection process, the surface of the brain organoid remained covered with the medium and a smooth pipette tip was used to prevent sliding of the brain organoid. The needle was maintained in the injection position for an additional 5 min and then slowly withdrawn to prevent cell leakage. After injection, the medium was replaced with fresh culture medium containing 10% penicillin–streptomycin, and the brain organoid was returned to the incubator. Fresh culture medium was replaced daily. The schematic diagram of organoid injection is shown in Fig. 1f. Each group included a minimum of three organoids.

### Mouse stereotaxic transplantation

Male NOD SCID mice (NOD.CB17-Prkdcscid/NCrCrl, Charles River, Wilmington, MA), aged 8–10 weeks, were used for stereotactic transplantation. All animal experiments followed guidelines set by the Institutional Animal Care and Use Committee of the Department of Laboratory Animal Science at Fudan University and approved by the Institutional Animal Care and Use Committee (Approval number: 202009007S). NOD SCID mice were anesthetized with 3% isoflurane at a flow rate of 1 l/min. Throughout the transplantation, anesthesia depth was monitored and adjusted to ensure a consistent level of anesthesia. Then 4 μl of cell suspension (50,000 cells/μl) was injected bilaterally into the striatum (AP: 0.5 mm, ML: ± 1.8 mm, DV: − 3.2 mm relative to bregma) of NOD SCID mice. The injection needle was maintained in the target position for 5 min and then slowly withdrawn over an additional 5 min. Following transplantation, the mice were monitored on a warm pad until recovery and were intraperitoneally administered with 0.9% sodium chloride for the next three days. Each group included a minimum of three mice.

### Brain sectioning and immunofluorescence (IF)

Mice were euthanized by perfusion with cold PBS and cold 4% paraformaldehyde (PFA), after which their brains were removed and post-fixed for an additional 2 h. The brains were further dehydrated and then embedded in paraffin wax. Paraffin blocks were sliced at a thickness of 6 μm to obtain coronal striatum sections. For IF staining of brain slices, the paraffin sections were deparaffined with xylene, followed by sequential rehydration in 100%, 95%, 70%, and 50% ethanol. The deparaffinized brain sections were subjected to antigen retrieval (Biosharp, Hefei, China) following the manufacturer’s instructions. The sections were then incubated three times with 0.3% PBST and blocked with 3% horse serum for 1 h. Subsequently, the sections were incubated with primary antibodies overnight at 4 °C, followed by incubation with immunofluorescence-conjugated secondary antibodies and Hoechst after washing away the primary antibodies. The resulting brain section images were visualized using a confocal microscope (Olympus FV3000). The antibodies used in this study are listed in Additional file 1: Table S1.

### Organoid sectioning and IF

Brain organoids were fixed in 4% cold PFA after harvesting. The following day, the brain organoids were sequentially cryoprotected in 20% sucrose and 30% sucrose solutions, embedded in optimal cutting temperature compound (OCT), and sectioned into 20-μm-thick slices. For IF staining, the sections were permeabilized in 0.3% PBST and blocked for one hour in 3% horse serum before adding a primary antibody. After incubating overnight with the primary antibody at 4 °C, the sections were washed with PBS, and incubated with a fluorophore-conjugated secondary antibody and Hoechst at room temperature for one hour, followed by a final wash with PBS. Images were captured using a confocal microscope (Olympus FV3000). The antibodies used in this study are listed in Additional file 1: Table S1.

### Immunohistochemistry (IHC)

Organoid sections were treated with 3% hydrogen peroxide to block endogenous peroxidase activity after permeabilization in 0.3% PBST. Then, the sections were incubated in 0.3% PBST supplemented with 3% BSA and 3% normal horse serum for one hour, followed by overnight incubation with a primary antibody at 4 °C. The following day, organoid sections were incubated with horseradish peroxidase (HRP)-conjugated secondary antibody for 1 h after washing with PBS. Next, the sections were visualized using the 3,3′-Diaminobenzidine (DAB) substrate application. Cell nuclei were counterstained with hematoxylin, followed by dehydration and mounting. Images were captured using a research slide scanner (Olympus VS200).

### Hematoxylin & eosin (H&E) staining

H&E staining was conducted according to the manufacturer’s guidelines (Yeasen, 60524ES60, Shanghai, China) and observed using a research slide scanner (Olympus VS200).

### Single-cell RNA sequencing

To analyze differential gene expression between cerebral organoids and GBM organoids, we performed single-cell RNA sequencing analysis using raw data obtained from the GEO database (GSE213835) of cerebral organoid and GBM organoid at 4 months of age [32]. Briefly, cells within 5%–95% of features in populations and with less than 10% of reads aligned to mitochondrial genes were selected for downstream analysis. Cell gene expression was normalized using a global-scaling normalization method and scaled using the Seurat package. Cell clusters were identified using the FindAllMarkers function in Seurat, after which cell types were classified based on reported markers of the human brain and its developmental stage [33, 34]. The Kyoto Encyclopedia of Genes and Genomes (KEGG) pathways enriched in defined clusters compared to other clusters were validated using Gene Set Enrichment Analysis.

### Statistical analysis

All data are presented as mean ± standard deviation (SD). The exact number of samples and the methods of statistical tests are provided in figure legends. All statistical analyses were performed using GraphPad Prism 8.0 (GraphPad Software). *P* < 0.05 was considered as statistically significant.

## Results

### Generation of mDA cells and cerebral organoids

Differentiation of mDA cells from hESCs was achieved following a protocol that incorporated dual SMAD inhibition (targeting BMP and TGF-β signaling) and dual activation of WNT and SHH pathways (Fig. 1a) [30, 31]. By day 21, the differentiated population was predominantly composed of midbrain dopamine progenitors (mDAPs), as demonstrated by high proportions of FOXA2^+^ (88.00% ± 2.00%), LMX1A^+^ (84.33% ± 6.11%), and EN1^+^ cells (85.33% ± 6.51%) (Fig. 1b, c). Additionally, a subset of cells were identified as mature dopamine neurons (TH^+^), constituting approximately 15.00% ± 3.00% of the population (Fig. 1b, c) and confirming effective differentiation toward mDA cells.

Cerebral organoids were generated following the Lancaster protocol [19, 35]. Neural progenitor markers Tuj-1, SOX2, and Nestin, and the immature neuronal marker doublecortin (DCX), exhibited robust expression in the cerebral organoids (Fig. 1e), indicating appropriate differentiation towards the cerebral lineage.

### Cerebral organoids substantially maintained the maturation of injected mDA cells and proliferation of injected hPSCs

**Fig. 2:**
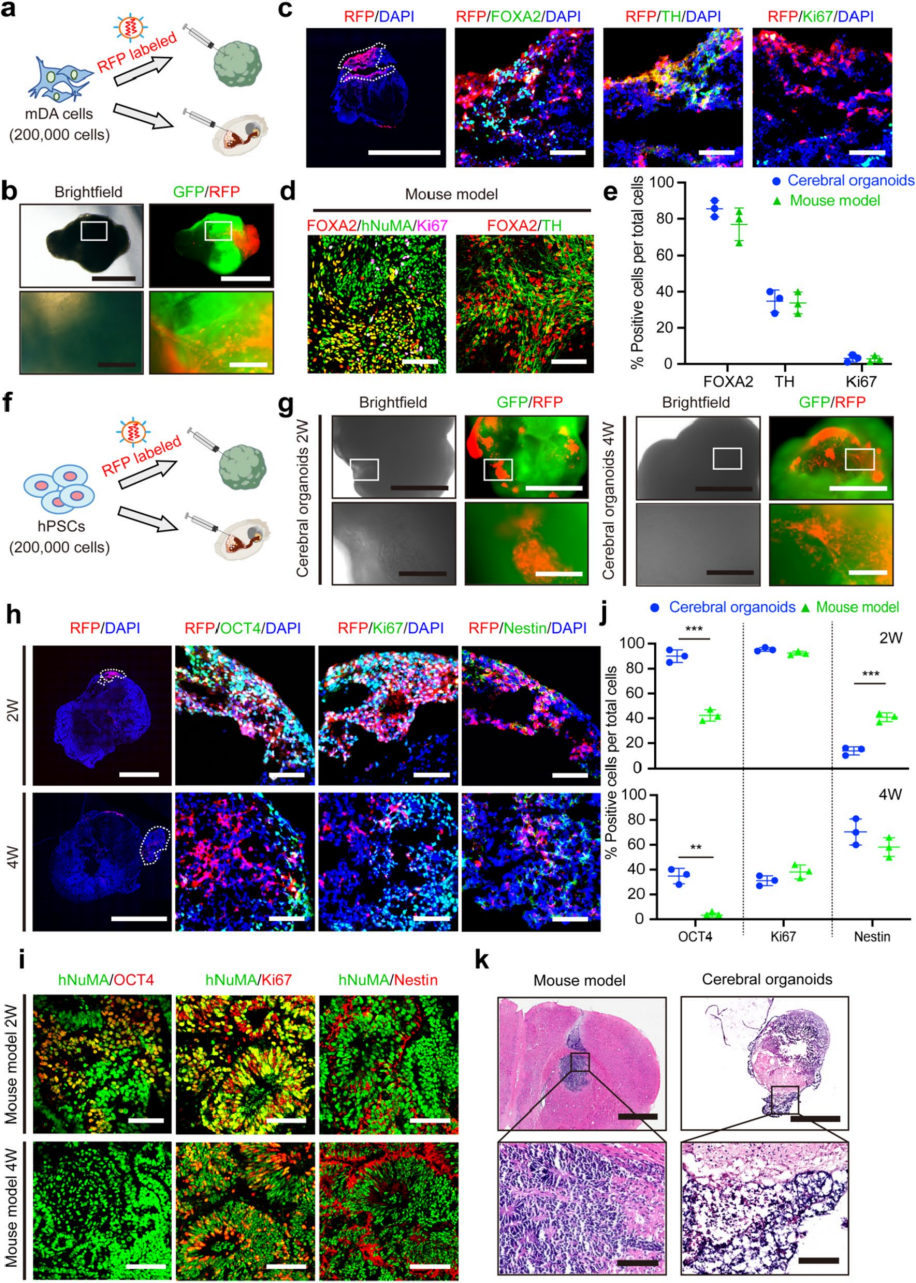
Cerebral organoids substantially maintained the maturation of injected mDA cells and proliferation of injected hPSCs. **a** mDA cells were labeled with RFP before injection into the cerebral organoids. **b** Bright-field and live images of mDA cells injected into the cerebral organoids at 4 weeks post-injection. Scale bars, upper 1000 μm, lower 200 μm. **c** Immunofluorescence of FOXA2, TH, and proliferating marker Ki67 in the cerebral organoids at 4 weeks after injection of mDA cells. Scale bars, leftmost 1000 μm, right three 80 μm. **d** Immunofluorescence images of grafted human cells (hNuMA), FOXA2, TH, and Ki67 in the NOD SCID mice at 4 weeks after transplantation of mDA cells. Scale bars: 80 μm. **e** Quantification of FOXA2^+^, TH^+^, and Ki67^+^ populations between the cerebral organoids and the mouse model at 4 weeks post injection. *n* = 3. **f** hPSCs were labeled with RFP before injection into the cerebral organoids. **g** The bright-field and live images of hPSCs injected into the cerebral organoids. Scale bars, upper 1000 μm, lower 200 μm. **h** Immunofluorescence of Nestin, Ki67, and pluripotent marker OCT4 after hPSC injection in the cerebral organoids. Scale bar: leftmost column 1000 μm, right columns 80 μm. **i** Immunofluorescence of hNuMA, OCT4, Nestin, and Ki67 of hPSCs after transplantation into the NOD SCID mice. Scale bars: 80 μm. **j** Quantification of OCT4^+^, Ki67^+^ and Nestin^+^ populations in the cerebral organoids and mouse model. *n* = 3. **k** H&E staining in the mouse model and the cerebral organoids at 4 weeks after injection of hPSCs. Scale bars: upper 1000 μm, lower 100 μm. For **e** and **j**, data are presented as mean ± SD, *n* = 3 for each group. Data were analyzed with two-tailed *t*-test

We microinjected 200,000 RFP-labeled mDA cells into the cerebral organoids (Fig. 2a). Meanwhile, we bilaterally transplanted the mDA cells into the striatum of NOD SCID mice. The characteristics of mDA cells in the cerebral organoids were compared to those in the in vivo condition.

The injected mDA cells demonstrated robust survival within the cerebral organoids, as indicated by sustained RFP expression for up to 12 weeks post-injection (Fig. 2b and Additional file 1: Fig. S1). Initially, the mDA cells were predominantly concentrated at the injection site. By 4 weeks, the mDA cells had extensively dispersed throughout the cerebral organoids, forming significant axonal bundles (Additional file 1: Fig. S1a, c, e). The majority of injected mDA cells in the cerebral organoids were positive for FOXA2 at four weeks (85.57% ± 4.50%) and 12 weeks (71.67% ± 7.10%) (Fig. 2c, e and Additional file 1: Fig. S1f, h). Additionally, a progressive increase was observed in the percentage of TH-positive cells over time in the cerebral organoids (4 weeks: 34.80% ± 6.16%; 12 weeks: 53.00% ± 6.56%). Notably, the expression levels of FOXA2 and TH were comparable between the cerebral organoids and animal model (Fig. 2d, e and Additional file 1: Fig. S1g, h).

To assess the safety profile of the injected mDA cells in the cerebral organoids, cell proliferation was analyzed by quantifying Ki67-positive cells and cellular morphology was evaluated by H&E staining. The mDA cells consistently showed low proliferative activity among at all time points examined within the cerebral organoids, with percentages of 3.10% ± 2.01% at 4 weeks and 3.23% ± 1.14% at 12 weeks post-injection for Ki67-positive cells (Fig. 2c, e and Additional file 1: Fig. S1f, h). Consistently, in vivo graft analysis showed comparable proliferation rates at corresponding time intervals (4 weeks: 2.83% ± 1.69%, 12 weeks: 2.71% ± 0.62%). Furthermore, H&E staining showed no evidence of abnormal nuclear morphology of mDA cells in the cerebral organoids (Additional file 1: Fig. S2).

Next, RFP-labeled hPSCs were microinjected into the cerebral organoid model (Fig. 2f). Unlike the mDA cells, most RFP-labeled hPSCs remained proximal around the injection sites within the cerebral organoids, forming numerous dense colonies. At two and four weeks post-injection, the hPSCs displayed progressive proliferation and invaded surrounding regions of the cerebral organoids (Fig. 2g and Additional file 1: Fig. S3). The progressive proliferation of injected hPSCs was further evident through immunofluorescence analysis. The majority of hPSCs showed expression of the proliferation marker Ki67, reaching a rate of 95.33% ± 1.53% at two weeks post-injection, which decreased to 31.10% ± 4.00% within four weeks post-injection (Fig. 2h–j). Nonetheless, there was no significant difference in the proliferative index of the injected hPSCs between the cerebral organoids and the mouse model. Moreover, most injected hPSCs retained expression of the pluripotency marker OCT4 after 2 weeks within the cerebral organoid model, with a significantly higher proportion than that observed in the animal model (*P* = 0.0003). The proportion of OCT4-positive hPSCs decreased to 34.80% ± 6.22% at 4 weeks post-injection but remained notably higher than that observed in the in vivo mouse model (*P* = 0.0010). The observed gradual reduction of proliferation and pluripotency of the injected hPSCs may be due to their differentiation into the neural lineage, as evidenced by the corresponding gradual increase in the expression of the neural precursor marker Nestin in the cerebral organoids. Ultimately, nuclear staining showed densely aggregated nuclei in both the mouse model and the cerebral organoids (Fig. 2k), indicating the preservation of the characteristic features of hPSCs.

In summary, the cerebral organoid model maintained the maturation and differentiation of mDA cells and supported extensive proliferation and maintained pluripotency of hPSCs. These suggest the potential of the cerebral organoid model as a fundamental platform for evaluating tumorigenic risk of PD cell therapies.

### Injected hPSCs exhibited an enhanced proliferative capacity within GBM organoids

**Fig. 3:**
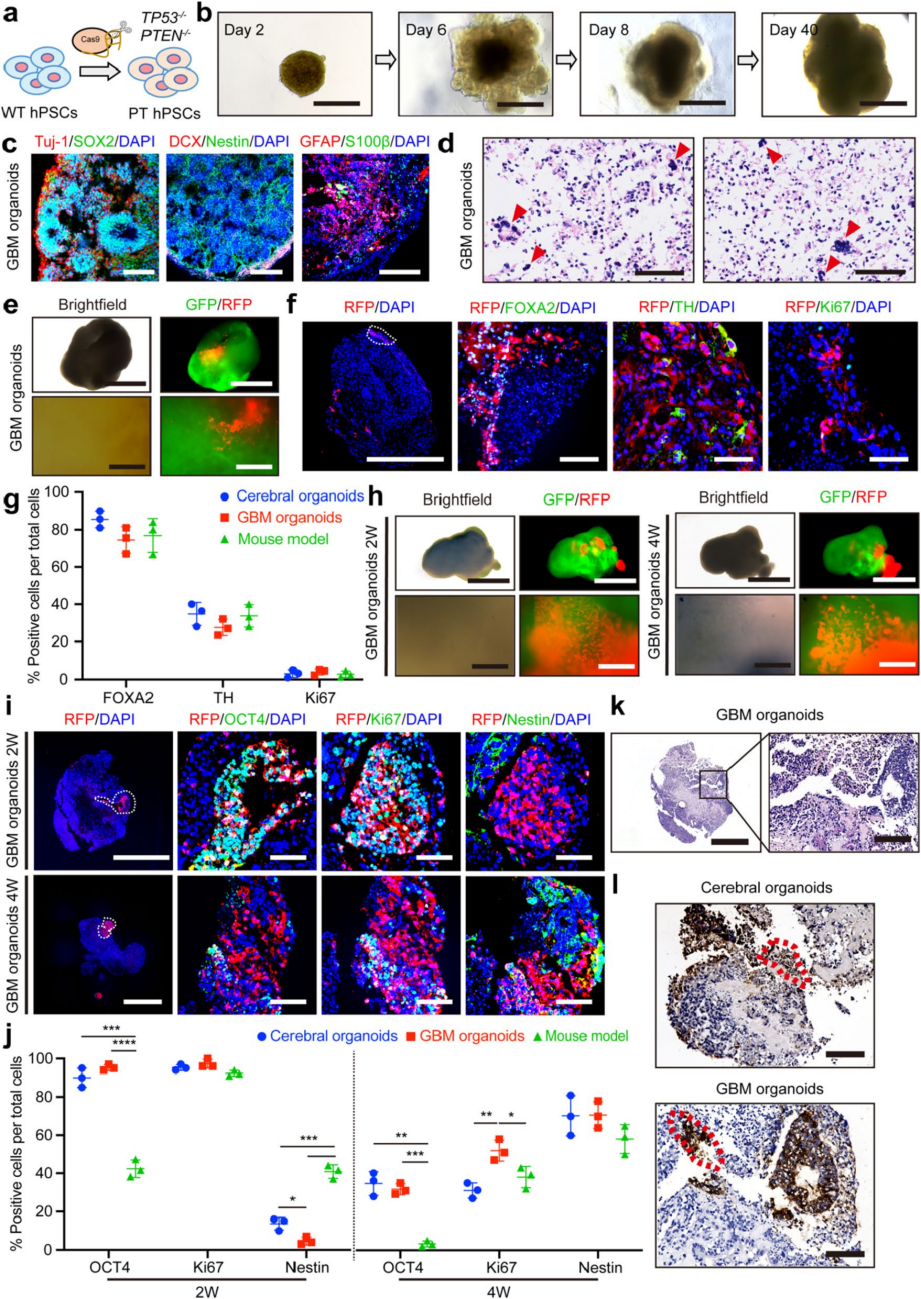
Injected hPSCs exhibited an enhanced proliferative capacity within GBM organoids. **a** Generation of GBM organoids from *TP*53 and *PTEN* mutant hPSCs (*TP*53^*−/−*^*/PTEN*^*−/−*^) using CRISPR-Cas9 technology. **b** Bright-field images of a GBM organoid. Scale bars: 1000 μm. **c** Immunofluorescence images of SOX2, Tuj-1, DCX, Nestin, and glial cells (GFAP/S100β) at day 30 of the GBM organoids. Scale bars: 200 μm. **d** H&E staining at day 30 of the GBM organoids. Scale bars: 100 μm. Red arrows indicate cells with atypical nuclei. **e** Bright-field and live images of mDA cells in the GBM organoids at 4 weeks post-injection. Scale bars, upper 1000 μm, lower 200 μm. **f** Immunofluorescence images of FOXA2, TH, and Ki67 in the GBM organoids at 4 weeks after injection of mDA cells. Scale bars: leftmost 1000 μm, right three 80 μm. **g** Quantification of FOXA2^+^, TH^+^, and Ki67^+^ populations in the cerebral organoids, GBM organoids and the mouse model at 4 weeks after injection of mDA cells. *n* = 3. **h** The bright-field and live images of hPSCs in the GBM organoids at 2 weeks and 4 weeks post-injection. Scale bars: upper 1000 μm, lower 200 μm. **i** Immunofluorescence images of OCT4, Ki67, and Nestin after hPSC injection into the GBM organoids. Scale bar: leftmost column 1000 μm, right columns 80 μm. **j** Quantification of OCT4^+^, Ki67^+^, and Nestin^+^ populations in the cerebral organoids, GBM organoids, and the mouse model. *n* = 3. **k** H&E staining of hPSCs injected into the GBM organoids. Scale bars: left 1000 μm, right 100 μm. **l** IHC staining of hPSCs after injection into the cerebral organoids and GBM organoids. Scale bars: 100 μm. The red dashed boxes highlight regions adjacent to the injection site, indicating migration of the injected cells. For **g** and **j**, data are presented as mean ± SD, *n* = 3 for each group. Data were analyzed with two-tailed *t*-test

A more sensitive and effective model is required for tumorigenicity evaluation. The tumor microenvironment (TME) can influence the phenotypic transformation and proliferation of various cell types, including astrocytes, adipose stem cells, and early NSCs [36–38]. Herein, we proposed providing a TME around the injected cells. As previous studies have reported that brain tumor organoids can simulate the TME [32, 39, 40], GBM organoids derived from *TP*53^*−/−*^*/PTEN*^*−/−*^ hPSCs were generated (Fig. 3a, b), expecting to exhibit increased sensitivity for detecting potentially unsafe cell products. In addition to exhibiting markers indicative of neural precursor cells, the GBM organoids were also distinguished by the expression of GFAP (glial fibrillary acidic protein) and S100β (Fig. 3c). Moreover, the GBM organoids displayed nuclear atypia, a characteristic sign of malignant transformation (Fig. 3d).

Like the mDA cells injected into the cerebral organoids, the mDA cells in the GBM organoids showed robust survival and developed long-distance axonal bundles at both 4 weeks and 12 weeks post-injection (Fig. 3e and Additional file 1: Fig. S1). The majority of injected mDA cells in the GBM organoids expressed FOXA2 at four weeks post-injection (74.55% ± 7.06%), and there was no significant difference in the proportion of FOXA2-positive mDA cells among the cerebral organoids, GBM organoids, and mouse model (Fig. 3f, g). Additionally, an increasing trend in the percentage of TH-positive cells was observed in the GBM organoids (4 weeks: 27.54% ± 4.36%, 12 weeks: 60.56% ± 2.68%). Notably, the mDA cells exhibited low proliferation rates within the GBM organoids (4 weeks: 4.03% ± 1.53%, 12 weeks: 5.31% ± 1.49%), indicating that the mDA cells were resistant to transitioning into a highly proliferative state, even within the potential tumorigenic microenvironment (Fig. 3f, g and Additional file 1: Fig. S1f, h).

In the GBM organoid model, injected hPSCs demonstrated remarkable survival, forming numerous colonies (Fig. 3h and Additional file 1: Fig. S3). Similar to the cerebral organoids, the injected hPSCs in the GBM organoids exhibited significantly enhanced pluripotency (OCT4 expression) compared to those in the mouse model (2 weeks, *P* < 0.0001; 4 weeks, *P* = 0.0001) (Fig. 3i, j). Notably, there was a significant difference in the proportion of proliferative hPSCs (Ki67 expression) between the cerebral organoids and GBM organoids. At two weeks post-injection, the majority of hPSCs in the GBM organoids expressed the proliferation marker Ki67 (97.33% ± 2.31%), comparable to those in cerebral organoids and the mouse model. At four weeks, although the proliferation rate of injected hPSCs in the GBM organoids declined to 52.03% ± 5.52%, it was significantly higher than that in the cerebral organoids (*P* = 0.0060) and the mouse model (*P* = 0.0377) (Fig. 3i, j). Furthermore, H&E staining showed deep aggregation of injected hPSCs in the GBM organoids (Fig. 3k). IHC analysis of hPSCs within the cerebral organoids and GBM organoids revealed their movement to adjacent areas as delineated by the red dashed lines in Fig. 3l, suggesting their migratory propensity. This migration indicates the robust survival of injected hPSCs and their high proliferative activity within the cerebral organoids and GBM organoids.

In summary, our data showed that the GBM organoids enhance the proliferative capacity of injected hPSCs while maintaining normal maturation of mDA cells. These findings suggest that the GBM organoid model is a favorable system for assessing the safety of cellular products.

### GBM organoids demonstrated an increased sensitivity in the detection of injected mDA cells spiked with hPSCs

In the therapeutic context, cell products might include residual hPSCs. Previous studies revealed that even a minimal residual population of 1% hPSCs is enough to initiate tumorigenesis [9, 11], thus highlighting the necessity for highly sensitive detection methods.

**Fig. 4:**
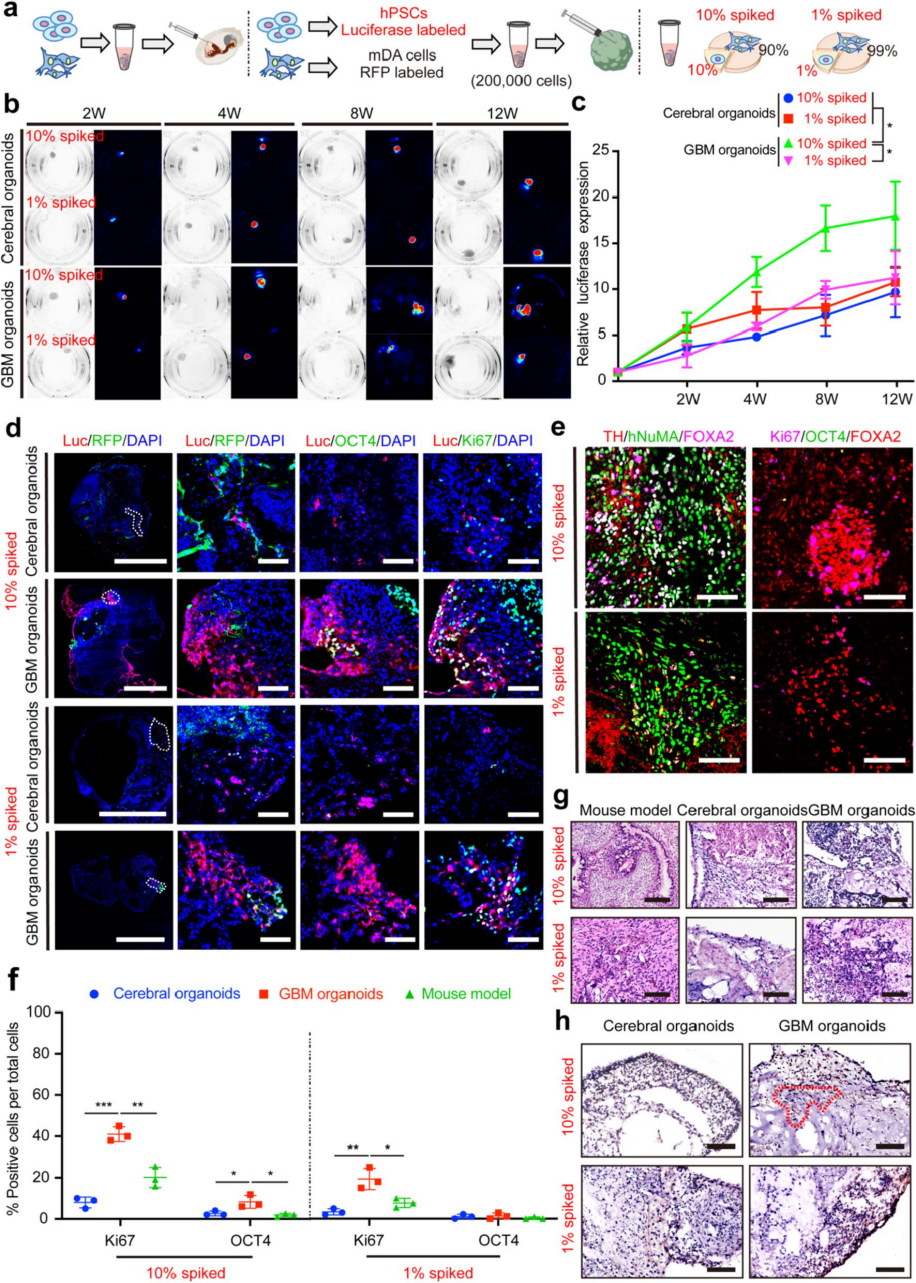
GBM organoids demonstrated an increased sensitivity in the detection of injected mDA cells spiked with hPSCs. **a** mDA cells were labeled with RFP, and hPSCs were labeled with luciferase. Subsequently, mDA cell suspensions containing 10% or 1% hPSCs were prepared for organoid injection. **b** The luciferase signal was visualized at 2, 4, 8, and 12 weeks post-injection. **c** Quantification of relative luciferase expression among different groups. The luciferase signals at different time points were normalized to the baseline level. Data are presented as mean ± SD, *n* = 5 in each group. Data were analyzed using one-way analysis of variance (ANOVA) followed by Dunnett’s multiple comparisons test. *: means significant difference, *P* < 0.05. **d** Immunofluorescence images of Luc/RFP, OCT4, and Ki67 in cerebral organoids and GBM organoids at 4 weeks after injection of mDA cells spiked with 10% or 1% hPSCs. Scale bars: leftmost column 1000 μm, right three columns 80 μm. **e** Immunofluorescence staining of hNuMA, FOXA2, TH, Ki67, and OCT4 in the striatum of NOD SCID mice at 4 weeks after transplantation of mDA cells spiked with hPSCs. Scale bars: 80 μm. **f** Quantification of Ki67^+^ and OCT4^+^ populations in the cerebral organoids, GBM organoids, and the mouse model at 4 weeks post injection. Data are presented as mean ± SD, *n* = 3 for each group. Data were analyzed using a two-tailed *t*-test. **g** H&E staining of mouse model, cerebral organoid and GBM organoid sections at 4 weeks post injection of spiked hPSCs. Scale bars: 100 μm. **h** IHC staining of cerebral organoids and GBM organoids at 4 weeks post injection of spiked hPSCs. Scale bars: 100 μm. The 10% hPSCs in the GBM organoids migrated to the region (red dashed box) adjacent to the injection site

Therefore, we assessed the abilities of cerebral organoids and GBM organoids to detect residual hPSCs within mDA cells. hPSCs were labeled with luciferase, and mDA cells were labeled with RFP. Cell suspension comprising 90% of mDA cells spiked with 10% hPSCs, or 99% mDA cells spiked with 1% hPSCs, was microinjected into the cerebral organoids and GBM organoids (Fig. 4a). Concurrently, mDA cells spiked with either 1% hPSCs or 10% hPSCs were also transplanted into the NOD SCID mice.

The mDA cells spiked with 1% or 10% hPSCs remained detectable and viable in cerebral organoids and GBM organoids for up to 12 weeks post-injection (Fig. 4b). Moreover, a time-dependent increase in luciferase signal intensity was observed, reaching a peak at 12 weeks post-injection (Fig. 4c). Notably, at 12 weeks after injection, there was a significantly higher luciferase signal of the 10% spiked group within the GBM organoids than within the cerebral organoids (*P* = 0.0352). Still, there was no significant difference in the luciferase signal intensity at 1% spiked hPSC between the cerebral organoids and the GBM organoids (*P* = 0.4857).

Regarding cell proliferation, a significantly increased percentage of Ki67-positive spiked hPSCs was observed in the GBM organoids (10% spiked: 41.00% ± 3.61%; 1% spiked: 19.33% ± 5.13%) compared to the cerebral organoids (10% spiked: 8.00% ± 2.65%, *P* = 0.0002; 1% spiked: 3.33% ± 1.53%, *P* = 0.0066) and the in vivo mouse model (10% spiked: 20.07% ± 4.89%, *P* = 0.0040; 1% spiked: 7.67% ± 2.26%, *P* = 0.0227) at 4 weeks after injection (Fig. 4d–f). In addition, regarding cell pluripotency, there was a significantly higher proportion of OCT4-positive hPSCs (8.27% ± 3.10%) within the GBM organoids at 10% spiked, compared to the cerebral organoids (2.67% ± 1.16%, *P* = 0.0427) and the mouse model (1.87% ± 0.81%, *P* = 0.0258) at 4 weeks post-injection. In contrast, the hPSCs at 1% showed a comparable level of pluripotency among cerebral organoids, GBM organoids and in vivo mouse grafts at 4 weeks after injection. Finally, H&E staining of hPSCs in brain organoids revealed pronounced deep nuclear staining in both cerebral organoids and GBM organoids, suggesting stem cell characteristics (Fig. 4g). It should be noted that residual hPSCs transplanted into the NSG mice [9] and athymic nude rats [11] could induce classical teratomas, whereas injected spiked hPSCs in brain organoids formed tumors with early stem cells. IHC staining revealed that the hPSCs at 10% exhibited enhanced migration to adjacent areas in the GBM organoids compared to those within the cerebral organoids (Fig. 4h).

Overall, the above results indicated that even a small proportion of hPSCs could be detected in both cerebral organoids and GBM organoids. Particularly, the spiked hPSCs in the GBM organoids demonstrated an enhanced proliferative capacity and pluripotency, suggesting that GBM organoids are a more sensitive evaluation platform for detecting residual hPSCs.

### GBM organoids showed increased proliferation of injected immature mDA cells

**Fig. 5:**
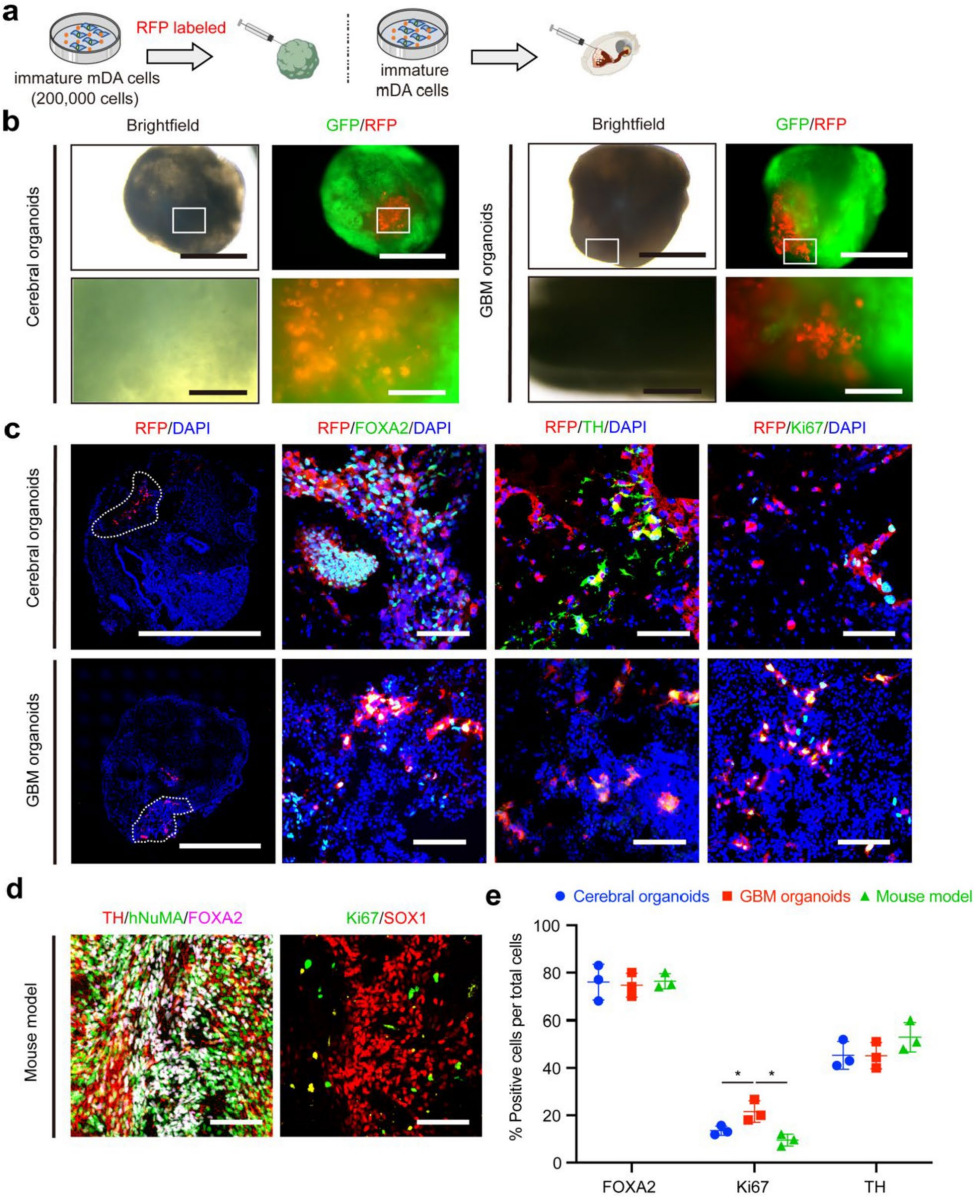
GBM organoids showed increased proliferation of injected immature mDA cells. **a** mDA cells at day 15 of differentiation were labeled with RFP before injection into the brain organoids. **b** The bright-field and live images of immature mDA cells after injection into the cerebral organoids and GBM organoids at 4 weeks. Scale bars, upper 1000 μm, lower 200 μm. **c** Immunofluorescence images of FOXA2, TH, and Ki67 staining of cerebral organoid and GBM organoid sections at 4 weeks after injection of immature mDA cells. Scale bars: leftmost column 1000 μm, right three columns 80 μm. **d** Immunofluorescence images of hNuMA, FOXA2, TH, Ki67, and SOX1 staining in NOD SCID mouse striatum sections at 4 weeks after transplantation of immature mDA cells. Scale bars: 80 μm. **e** Quantification of FOXA2^+^, Ki67^+^, and TH^+^ populations in the cerebral organoids, GBM organoids, and the mouse model at 4 weeks after injection of immature mDA cells. Data are presented as mean ± SD, *n* = 3 for each group. Data were analyzed with two-tailed *t*-test

Immature, undifferentiated neural progenitor cells within the final cell products are another crucial risk factor for tumorigenesis [41–44]. For instance, approximately 40% of the grafted immature mDA cells display the emergence of rosette-like structures [30]. Therefore, to elucidate the detection capabilities of brain organoids for these immature neural progenitors, mDA cells at day 15 of differentiation were selected as immature mDA cells (Fig. 5a).

The immature mDA cells exhibited substantial survival within both cerebral organoids and GBM organoids (Fig. 5b). The majority of injected cells within the cerebral organoids (76.00% ± 7.55%) and GBM organoids (74.67% ± 5.03%) were positive for the mDAP marker FOXA2, consistent with findings from grafts in the mouse model (76.33% ± 3.22%) (Fig. 5c–e). Noteworthy, we observed a significant increase in the proliferation of immature mDA cells in the GBM organoids (21.57% ± 4.56%) compared to the cerebral organoids (13.54% ± 1.96%, *P* = 0.0486) and the in vivo mouse model (9.56% ± 2.50%, *P* = 0.0161).

These findings indicated that the GBM organoids have superior sensitivity in detecting undifferentiated neural progenitors compared to the cerebral organoids and the animal model.

### Enhanced tumor-related metabolic pathways and cytokines may contribute to the elevated evaluation sensitivity of GBM organoids

To evaluate the mechanisms underlying the improved detection of tumorigenesis in the GBM organoids compared to the cerebral organoids, we re-analyzed the previously published single-cell RNA sequencing data from both cerebral organoids and GBM organoids [32].

**Fig. 6:**
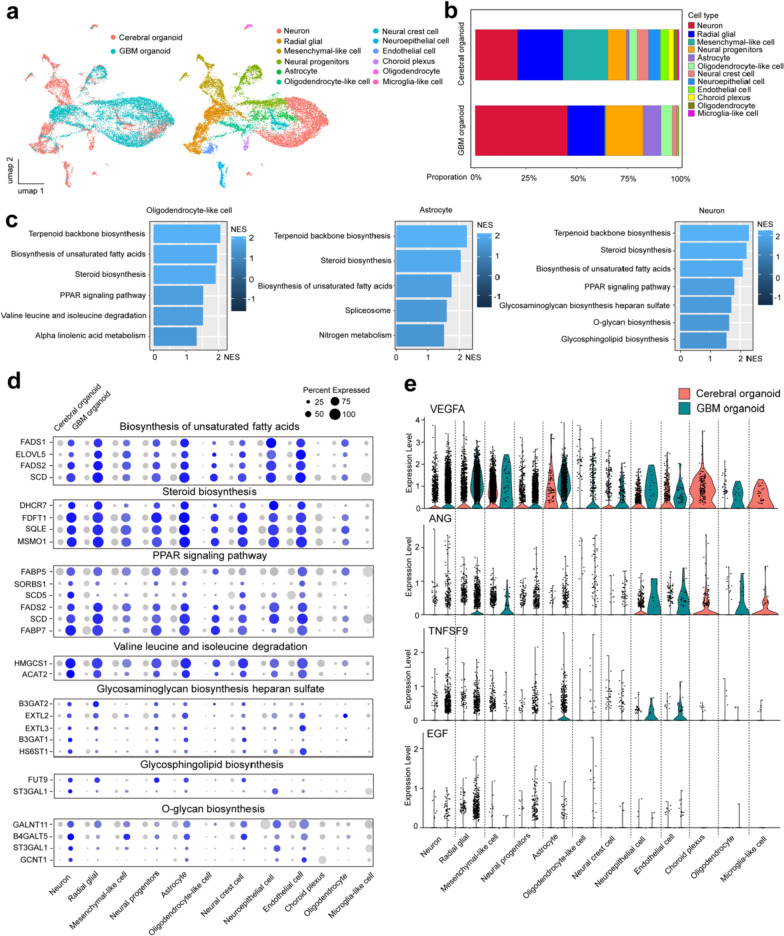
Enhanced tumor-related metabolic pathways and cytokines may contribute to the elevated evaluation sensitivity of GBM organoids. **a** Visualization of single-cell clustering in the cerebral organoids and GBM organoids using Uniform Manifold Approximation and Projection (u-map), colored by group and by cell type. *n* = 7090 (cerebral organoid) and* n* = 7706 (GBM organoid) cells from 2 organoids, respectively. **b** Proportions of cell types in the cerebral organoids and GBM organoids. **c** Top KEGG pathways for genes enriched in the cell types “oligodendrocyte-like cell”, “astrocyte”, and “neuron” compared to other cell types. **d** Bubble charts showing significantly differentially expressed genes in metabolic pathways for each cell type in cerebral organoids and GBM organoids. **e** Violin plots of selected cytokines for each cell type in cerebral organoids and GBM organoids

We discovered that cells in cerebral organoids were grouped into 12 clusters, while cells in GBM organoids were grouped into 10 clusters. These clusters were further analyzed based on reported markers of the human brain and its developmental stages [33, 34]. Compared to cerebral organoids, GBM organoids exhibited a higher presence of neurons, neural progenitors, astrocytes, and oligodendrocyte-like cells (Fig. 6a, b). KEGG analysis revealed that various tumor-related metabolic pathways were significantly upregulated in GBM organoid, including lipid metabolism (biosynthesis of unsaturated fatty acids, steroid biosynthesis, PPAR signaling pathway), amino acid metabolism (valine leucine and isoleucine degradation), glycan metabolism (glycosphingolipid biosynthesis, heparan sulfate biosynthesis, O-glycan biosynthesis) and terpenoids metabolism (Terpenoid backbone biosynthesis) (Fig. 6c, d). Furthermore, several pro-tumor cytokines, including VEGFA (Vascular Endothelial Growth Factor A), ANG (Angiogenin), TNFSF9 (TNF Superfamily Member 9), and EGF (Epidermal Growth Factor), were significantly increased in GBM organoids (Fig. 6e). Previous studies have shown that the TME dictates the maintenance of cancer stem cells or cancer stem-like cells to arbitrate cancer progression [45]. Here, we propose that the TME, including the elevated tumor-related metabolic pathways and cytokines detected in GBM organoids, contributes to the increased sensitivity of GBM organoids for safety evaluation.

## Discussion

**Fig. 7:**
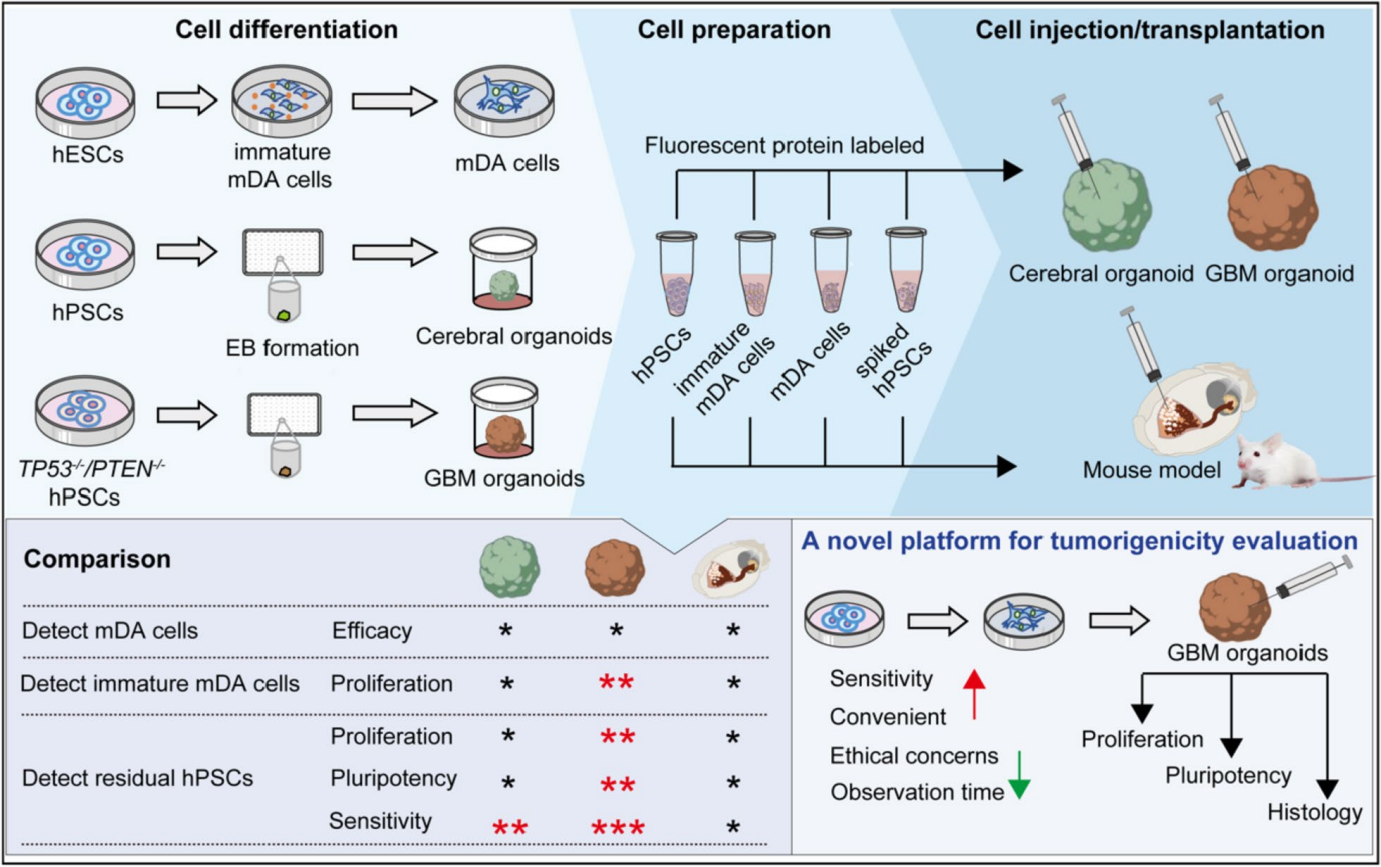
Schematic of this study. Both cerebral organoids and GBM organoids present promising and sensitive approaches for evaluating tumorigenicity in PD cell therapies. Importantly, the GBM organoids offer an enhanced model for detecting residual hPSCs and immature neural progenitors, underscoring its potential as a superior platform in tumorigenesis studies

In this study, we found that: (1) both cerebral organoids and GBM organoids maintained the maturation of injected mDA cells; (2) the GBM organoids exhibited a greater proliferative capacity of injected hPSCs compared to the cerebral organoids and the mouse model; (3) a small portion of hPSCs within cell products could be detected in brain organoids, particularly in GBM organoids that demonstrated a superior ability to enhance the proliferation and pluripotency of spiked hPSCs; (4) the GBM organoids displayed an increased proliferative ability for injected immature mDA cells; and (5) the high detection sensitivity of the GBM organoids may be attributed to the enhanced tumor-related metabolic pathways and cytokines. Overall, the GBM organoids provide a valuable platform for detecting residual hPSCs and immature progenitors with high sensitivity (Fig. 7).

Compared to traditional tumorigenicity evaluation using immunocompromised rodent models, brain organoids present several advantages.

1. Improved detection sensitivity. The increased proliferative capacity and pluripotency of residual hPSCs (Fig. 4) and immature mDA cells (Fig. 5) within GBM organoids imply that these risk cells have higher survival rates in GBM organoids, thereby enhancing the detection sensitivity for potential tumorigenic cells. Additionally, a spiking study [9] found that all mice grafted with MSK-DA01 spiked with 10% hPSCs and 33.3% of the mice with 1% hPSCs formed teratomas. Similarly, a Korean preclinical study [11] detected tumor formation in 30% of animals receiving TED-A9 spiked with 10% hPSCs and 15% of mice with 1% hPSCs. In contrast, our results showed that all cerebral organoids and GBM organoids exhibited detectable signals when the mDA cells were spiked with 10% and 1% hPSCs. The increased detection rates of hPSCs indicate the superior sensitivity of brain organoids for screening potential risk cells in cell products.
2. Reduced observation time. Animal-based tumorigenicity evaluations typically require long-term monitoring, lasting from months to years [9, 11, 12], which may cause a delay of application of potential treatments for individuals with neurodegenerative disorders. Nonetheless, the increased sensitivity of brain organoids for detecting risk cells could significantly reduce the time required for tumorigenicity evaluation.
3. Enhanced convenience and accessibility. Brain organoids offer a more straightforward procedure from organoid injection to sample collection, compared to mouse models.
4. Reduced ethical concerns. Animal studies are subject to significant ethical considerations due to the prolonged observation periods and the use for numerous animals. However, brain organoids present fewer ethical concerns in safety studies, making them a more ethically acceptable alternative for research [46, 47].

Nevertheless, brain organoids in this study have several limitations that warrant further investigation. First, it is essential to identify the most suitable brain organoid models that accurately reflect the composition and environment of patients’ brains. The cerebral organoid and GBM organoid models used in our study both have limitations in promoting neuronal maturation, as they primarily consisted of early NSCs and lacked blood vessels and immune cells, which are different from human brains. Therefore, it is necessary to develop vascularized brain organoids, or those with more diverse cell compositions and mature neurons, or patient-derived organoids that better replicate the brain composition and environment in PD patients. The emerging striatum organoids [48, 49], midbrain organoids [50–52], midbrain-striatum-cortex assembloids [29], brain organoids with microglia [53], and vascularized organoids [54, 55] should be tested to identify the most suitable brain organoid model for assessing tumorigenic potential. Second, while residual hPSCs demonstrated the ability to induce tumor formation in the animal model (Fig. 4g), their propensity to form the classical phenotype of tumors within brain organoids was less pronounced. Transplantation of brain organoids into rodents provides an opportunity to further investigate the tumorigenic behavior of residual hPSCs in brain organoids.

For future clinical translation of stem cell-based therapies, we recommend integrating GBM organoids as an innovative tumorigenicity evaluation tool to supplement or even substitute conventional animal-based tests. Compared to the cerebral organoids, the GBM organoids offer several advantages. (1) GBM organoids exhibit faster growth and greater extensivity than cerebral organoids [32]. The larger size of GBM organoids provides greater stability in the evaluation system and reduces technical complexities, enhancing their potential for future translational applications. (2) The tumor-related metabolic pathways and cytokines in the GBM organoids contribute to higher sensitivity in detecting potential risk cells, thereby improving the safety of cell therapies. Recent evidence suggests that TME dictates the cancer stem cells or cancer stem-like cells maintenance to arbitrate cancer progression [45]. Specially, elevated lipid metabolism facilitates the production of metabolites and lipid-related signaling molecules, such as cytokines and bioactive lipids, which modulate the microenvironment and supply energy to adjacent cells [56, 57]. Valine, leucine, and isoleucine, which are branched-chain amino acids, undergo degradation to generate metabolites, including glutamate, that are integrated into other metabolic pathways essential for tumorigenesis [58]. Glycans can attach to proteins and lipids to form glycoconjugates, including glycosphingolipid, heparan sulfate and O-glycan. These glycoconjugates exert pivotal roles in cancer biology through cell signaling, cell–matrix interactions, tumor angiogenesis, and metastasis, driven by alterations in glycosylation [59]. Additionally, pro-tumor cytokines play a crucial role in cell communication within the microenvironment, and abnormal generation of cytokines leads to downstream oncogenic changes in both malignant and nonmalignant cells [60, 61]. Herein, we propose that the TME in the GBM organoids may  contribute to the maintenance of  the high proliferation of risk cells.

We proposed a protocol for GBM organoid-based tumorigenicity evaluation (Fig. 7). Cell suspensions of positive control hPSCs, differentiated mDA cells, differentiated mDA cells spiked with 10% hPSCs, and differentiated mDA cells spiked with 1% hPSCs should be carefully prepared and injected into the GBM organoids. Follow-up evaluations are critical at 4 and 12 weeks post-injection, focusing on assessments of cell proliferation, cell pluripotency, and histological examination. Particular attention should be paid to signs of tumorigenicity risk, including the presence of pluripotent cells, increased proportions of proliferating cells, densely stained nuclei, and signs of cellular migration.

Stem cell-based therapies are being developed for various central nervous system (CNS) disorders, including injuries (spinal cord and traumatic brain injury), strokes, neurodegenerative diseases (PD, Alzheimer’s disease, retinal degeneration, amyotrophic lateral sclerosis), and epilepsy [62]. Safety is regarded as the primary endpoint in most clinical trials involving transplantation of hPSC-derived neurons [63]. However, patients often need to wait a long time to receive cell products, as final cells must undergo strict assessment by regulatory agencies. In this study, we confirmed that brain organoids could serve as a novel platform for evaluating both tumorigenicity and efficacy in PD cell therapies. These findings further suggest that brain organoids could be used as a novel quality control (QC) platform assessing safety and efficacy of stem cell-based therapies for CNS disorders. On the one hand, its high sensitivity may reduce evaluation time, allowing earlier administration of cell therapies in patients. On the other hand, brain organoids provide a pre-clinical evaluation platform for some CNS disorders that lack adequate animal models. Future research should focus on identifying the most appropriate organoid models that closely mirror the environment and the neuronal composition of patients’ brains. Notably, patient-derived brain organoids can serve as a more precise tool for assessing the safety and efficacy of cell therapies, enabling better evaluation of potential safety issues and therapeutic responses. This approach offers a new technological avenue for precision and personalized treatment.

## Conclusions

The present study explored the potential of brain organoids as an innovative platform for assessing tumorigenic potentials of stem cell-based therapies. Both cerebral organoids and GBM organoids offer a promising and sensitive approach for evaluating tumorigenicity in PD cell therapies. Notably, the GBM organoids had superior performance in detecting residual hPSCs and immature mDA cells, thus underscoring its potential use as a superior tool in tumorigenesis studies.

## Supplementary Information

**Additional file 1**: **Fig. S1** The long-term cell fate of mDA cells after being injected into the brain organoids. **Fig. S2** H&E staining of brain organoids and mouse brain sections after mDA cells injection. **Fig. S3** Live images of brain organoids after hPSCs injection. **Table S1**: Antibodies used in this study

## Data Availability

All data generated or analyzed during this study are included in this article and Additional file 1 or are available from the corresponding author on reasonable request. This study did not generate new unique reagents.

## Abbreviations

CNS: Central nervous system
EB: Embryoid body
GBM: Glioblastoma
hESCs: Human embryonic stem cells
hPSCs: Human pluripotent stem cells
KEGG: Kyoto encyclopedia of genes and genomes
mDA: Midbrain dopamine
mDAPs: Midbrain dopamine progenitors
NSCs: Neural stem cells
PD: Parkinson’s disease
TME: Tumor microenvironment

## References

[1] Yamanaka S. Pluripotent stem cell-based cell therapy-promise and challenges. Cell Stem Cell. 2020;27(4):523–31. 33007237 10.1016/j.stem.2020.09.014

[2] Temple S. Advancing cell therapy for neurodegenerative diseases. Cell Stem Cell. 2023;30(5):512–29. 37084729 10.1016/j.stem.2023.03.017 PMC10201979

[3] Goldring CE, Duffy PA, Benvenisty N, Andrews PW, Ben-David U, Eakins R, et al. Assessing the safety of stem cell therapeutics. Cell Stem Cell. 2011;8(6):618–28. 21624806 10.1016/j.stem.2011.05.012

[4] Lee AS, Tang C, Rao MS, Weissman IL, Wu JC. Tumorigenicity as a clinical hurdle for pluripotent stem cell therapies. Nat Med. 2013;19(8):998–1004. 23921754 10.1038/nm.3267 PMC3967018

[5] Lovell-Badge R, Anthony E, Barker RA, Bubela T, Brivanlou AH, Carpenter M, et al. ISSCR guidelines for stem cell research and clinical translation: the 2021 UPDATE. Stem Cell Rep. 2021;16(6):1398–408. 10.1016/j.stemcr.2021.05.012 PMC8190668 34048692

[6] Mayor S. First patient enters trial to test safety of stem cells in spinal injury. BMJ. 2010;341: c5724. 20940208 10.1136/bmj.c5724

[7] Wise J. Stroke patients take part in “milestone” UK trial of stem cell therapy. BMJ. 2010;341: c6574. 21081623 10.1136/bmj.c6574

[8] Schweitzer JS, Song B, Herrington TM, Park TY, Lee N, Ko S, et al. Personalized iPSC-derived dopamine progenitor cells for Parkinson’s disease. N Engl J Med. 2020;382(20):1926–32. 32402162 10.1056/NEJMoa1915872 PMC7288982

[9] Piao J, Zabierowski S, Dubose BN, Hill EJ, Navare M, Claros N, et al. 2021 Preclinical efficacy and safety of a human embryonic stem cell-derived midbrain dopamine progenitor product, MSK-DA01. Cell Stem Cell. 2021;28(2):217–29. 33545080 10.1016/j.stem.2021.01.004 PMC7903922

[10] Doi D, Magotani H, Kikuchi T, Ikeda M, Hiramatsu S, Yoshida K, et al. Pre-clinical study of induced pluripotent stem cell-derived dopaminergic progenitor cells for Parkinson’s disease. Nat Commun. 2020;11(1):3369. 32632153 10.1038/s41467-020-17165-w PMC7338530

[11] Park S, Park CW, Eom JH, Jo MY, Hur HJ, Choi SK, et al. Preclinical and dose-ranging assessment of hESC-derived dopaminergic progenitors for a clinical trial on Parkinson’s disease. Cell Stem Cell. 2024;31(1):25–38. 38086390 10.1016/j.stem.2023.11.009

[12] Kirkeby A, Nelander J, Hoban DB, Rogelius N, Bjartmarz H, Novo Nordisk Cell Therapy R, et al. Preclinical quality, safety, and efficacy of a human embryonic stem cell-derived product for the treatment of Parkinson’s disease STEM-PD. Cell Stem Cell. 2023;30(10):1299–314. 37802036 10.1016/j.stem.2023.08.014

[13] Cyranoski D. Strange lesions after stem-cell therapy. Nature. 2010;465(7301):997. 20577182 10.1038/465997a

[14] Amariglio N, Hirshberg A, Scheithauer BW, Cohen Y, Loewenthal R, Trakhtenbrot L, et al. Donor-derived brain tumor following neural stem cell transplantation in an ataxia telangiectasia patient. PLoS Med. 2009;6(2): e1000029. 19226183 10.1371/journal.pmed.1000029 PMC2642879

[15] Han L, He H, Yang Y, Meng Q, Ye F, Chen G, et al. Distinctive clinical and pathologic features of immature teratomas arising from induced pluripotent stem cell-derived beta cell injection in a diabetes patient. Stem Cells Dev. 2022;31(5–6):97–101. 35018826 10.1089/scd.2021.0255

[16] Masjosthusmann S, Becker D, Petzuch B, Klose J, Siebert C, Deenen R, et al. A transcriptome comparison of time-matched developing human, mouse and rat neural progenitor cells reveals human uniqueness. Toxicol Appl Pharmacol. 2018;354:40–55. 29753005 10.1016/j.taap.2018.05.009

[17] Hodge RD, Bakken TE, Miller JA, Smith KA, Barkan ER, Graybuck LT, et al. Conserved cell types with divergent features in human versus mouse cortex. Nature. 2019;573(7772):61–8. 31435019 10.1038/s41586-019-1506-7 PMC6919571

[18] Garitaonandia I, Gonzalez R, Christiansen-Weber T, Abramihina T, Poustovoitov M, Noskov A, et al. Neural stem cell tumorigenicity and biodistribution assessment for phase i clinical trial in Parkinson’s disease. Sci Rep. 2016;6:34478. 27686862 10.1038/srep34478 PMC5055076

[19] Lancaster MA, Renner M, Martin CA, Wenzel D, Bicknell LS, Hurles ME, et al. Cerebral organoids model human brain development and microcephaly. Nature. 2013;501(7467):373–9. 23995685 10.1038/nature12517 PMC3817409

[20] Chiaradia I, Lancaster MA. Brain organoids for the study of human neurobiology at the interface of in vitro and in vivo. Nat Neurosci. 2020;23(12):1496–508. 33139941 10.1038/s41593-020-00730-3

[21] Qian X, Song H, Ming GL. Brain organoids: advances, applications and challenges. Development. 2019;146(8):dev166074. 30992274 10.1242/dev.166074 PMC6503989

[22] Kelley KW, Pasca SP. Human brain organogenesis: Toward a cellular understanding of development and disease. Cell. 2022;185(1):42–61. 34774127 10.1016/j.cell.2021.10.003 PMC13523617

[23] Pasca AM, Sloan SA, Clarke LE, Tian Y, Makinson CD, Huber N, et al. Functional cortical neurons and astrocytes from human pluripotent stem cells in 3D culture. Nat Methods. 2015;12(7):671–8. 26005811 10.1038/nmeth.3415 PMC4489980

[24] Luo C, Lancaster MA, Castanon R, Nery JR, Knoblich JA, Ecker JR. Cerebral organoids recapitulate epigenomic signatures of the human fetal brain. Cell Rep. 2016;17(12):3369–84. 28009303 10.1016/j.celrep.2016.12.001 PMC5495578

[25] Lancaster MA, Knoblich JA. Organogenesis in a dish: modeling development and disease using organoid technologies. Science. 2014;345(6194):1247125. 25035496 10.1126/science.1247125

[26] Amin ND, Pasca SP. Building models of brain disorders with three-dimensional organoids. Neuron. 2018;100(2):389–405. 30359604 10.1016/j.neuron.2018.10.007

[27] Garcia-Delgado AB, Campos-Cuerva R, Rosell-Valle C, Martin-Lopez M, Casado C, Ferrari D, et al. Brain organoids to evaluate cellular therapies. Animals (Basel). 2022;12(22):3150. 36428378 10.3390/ani12223150 PMC9686900

[28] Ibanez-Rios MI, Narlis M, Hammond CA, Knapp D. A human cerebral organoid model of neural cell transplantation. J Vis Exp. 2023. 10.3791/64770. 37590518 10.3791/64770

[29] Reumann D, Krauditsch C, Novatchkova M, Sozzi E, Wong SN, Zabolocki M, et al. In vitro modeling of the human dopaminergic system using spatially arranged ventral midbrain-striatum-cortex assembloids. Nat Methods. 2023;20(12):2034–47. 38052989 10.1038/s41592-023-02080-x PMC10703680

[30] Song B, Cha Y, Ko S, Jeon J, Lee N, Seo H, et al. Human autologous iPSC-derived dopaminergic progenitors restore motor function in Parkinson’s disease models. J Clin Invest. 2020;130(2):904–20. 31714896 10.1172/JCI130767 PMC6994130

[31] Kim J, Jeon J, Song B, Lee N, Ko S, Cha Y, et al. Spotting-based differentiation of functional dopaminergic progenitors from human pluripotent stem cells. Nat Protoc. 2022;17(3):890–909. 35140411 10.1038/s41596-021-00673-4 PMC9511827

[32] Wang C, Sun M, Shao C, Schlicker L, Zhuo Y, Harim Y, et al. A multidimensional atlas of human glioblastoma-like organoids reveals highly coordinated molecular networks and effective drugs. NPJ Precis Oncol. 2024;8(1):19. 38273014 10.1038/s41698-024-00500-5 PMC10811239

[33] Lake BB, Ai R, Kaeser GE, Salathia NS, Yung YC, Liu R, et al. Neuronal subtypes and diversity revealed by single-nucleus RNA sequencing of the human brain. Science. 2016;352(6293):1586–90. 27339989 10.1126/science.aaf1204 PMC5038589

[34] Kanton S, Boyle MJ, He Z, Santel M, Weigert A, Sanchis-Calleja F, et al. Organoid single-cell genomic atlas uncovers human-specific features of brain development. Nature. 2019;574(7778):418–22. 31619793 10.1038/s41586-019-1654-9

[35] Lancaster MA, Knoblich JA. Generation of cerebral organoids from human pluripotent stem cells. Nat Protoc. 2014;9(10):2329–40. 25188634 10.1038/nprot.2014.158 PMC4160653

[36] Gagliano N, Costa F, Cossetti C, Pettinari L, Bassi R, Chiriva-Internati M, et al. Glioma-astrocyte interaction modifies the astrocyte phenotype in a co-culture experimental model. Oncol Rep. 2009;22(6):1349–56. 19885586 10.3892/or_00000574

[37] Farrell K, Mahajan G, Srinivasan P, Lee MY, Kothapalli CR. Pediatric glioblastoma cells inhibit neurogenesis and promote astrogenesis, phenotypic transformation and migration of human neural progenitor cells within cocultures. Exp Cell Res. 2018;362(1):159–71. 29129566 10.1016/j.yexcr.2017.11.013 PMC5741502

[38] Paino F, La Noce M, Di Nucci D, Nicoletti GF, Salzillo R, De Rosa A, et al. Human adipose stem cell differentiation is highly affected by cancer cells both in vitro and in vivo: implication for autologous fat grafting. Cell Death Dis. 2017;8(1): e2568. 28102844 10.1038/cddis.2016.308 PMC5386348

[39] Linkous A, Balamatsias D, Snuderl M, Edwards L, Miyaguchi K, Milner T, et al. Modeling patient-derived glioblastoma with cerebral organoids. Cell Rep. 2019;26(12):3203–11. 30893594 10.1016/j.celrep.2019.02.063 PMC6625753

[40] Wen J, Liu F, Cheng Q, Weygant N, Liang X, Fan F, et al. Applications of organoid technology to brain tumors. CNS Neurosci Ther. 2023;29(10):2725–43. 37248629 10.1111/cns.14272 PMC10493676

[41] Doi D, Morizane A, Kikuchi T, Onoe H, Hayashi T, Kawasaki T, et al. Prolonged maturation culture favors a reduction in the tumorigenicity and the dopaminergic function of human ESC-derived neural cells in a primate model of Parkinson’s disease. Stem Cells. 2012;30(5):935–45. 22328536 10.1002/stem.1060

[42] Brederlau A, Correia AS, Anisimov SV, Elmi M, Paul G, Roybon L, et al. Transplantation of human embryonic stem cell-derived cells to a rat model of Parkinson’s disease: effect of in vitro differentiation on graft survival and teratoma formation. Stem Cells. 2006;24(6):1433–40. 16556709 10.1634/stemcells.2005-0393

[43] Katsukawa M, Nakajima Y, Fukumoto A, Doi D, Takahashi J. Fail-safe therapy by gamma-ray irradiation against tumor formation by human-induced pluripotent stem cell-derived neural progenitors. Stem Cells Dev. 2016;25(11):815–25. 27059007 10.1089/scd.2015.0394

[44] Moon J, Lee HS, Kang JM, Park J, Leung A, Hong S, et al. Stem cell grafting improves both motor and cognitive impairments in a genetic model of Parkinson’s disease, the aphakia (ak) mouse. Cell Transplant. 2013;22(7):1263–79. 23031199 10.3727/096368912X657242 PMC3752607

[45] Nallasamy P, Nimmakayala RK, Parte S, Are AC, Batra SK, Ponnusamy MP. Tumor microenvironment enriches the stemness features: the architectural event of therapy resistance and metastasis. Mol Cancer. 2022;21(1):225. 36550571 10.1186/s12943-022-01682-x PMC9773588

[46] Sawai T, Sakaguchi H, Thomas E, Takahashi J, Fujita M. The ethics of cerebral organoid research: being conscious of consciousness. Stem Cell Reports. 2019;13(3):440–7. 31509736 10.1016/j.stemcr.2019.08.003 PMC6739740

[47] Jeziorski J, Brandt R, Evans JH, Campana W, Kalichman M, Thompson E, et al. Brain organoids, consciousness, ethics and moral status. Semin Cell Dev Biol. 2023;144:97–102. 35339359 10.1016/j.semcdb.2022.03.020

[48] Miura Y, Li MY, Birey F, Ikeda K, Revah O, Thete MV, et al. Generation of human striatal organoids and cortico-striatal assembloids from human pluripotent stem cells. Nat Biotechnol. 2020;38(12):1421–30. 33273741 10.1038/s41587-020-00763-w PMC9042317

[49] Miura Y, Li MY, Revah O, Yoon SJ, Narazaki G, Pasca SP. Engineering brain assembloids to interrogate human neural circuits. Nat Protoc. 2022;17(1):15–35. 34992269 10.1038/s41596-021-00632-z

[50] Qian X, Jacob F, Song MM, Nguyen HN, Song H, Ming GL. Generation of human brain region-specific organoids using a miniaturized spinning bioreactor. Nat Protoc. 2018;13(3):565–80. 29470464 10.1038/nprot.2017.152 PMC6241211

[51] Jo J, Xiao Y, Sun AX, Cukuroglu E, Tran HD, Goke J, et al. Midbrain-like organoids from human pluripotent stem cells contain functional dopaminergic and neuromelanin-producing neurons. Cell Stem Cell. 2016;19(2):248–57. 27476966 10.1016/j.stem.2016.07.005 PMC5510242

[52] Fiorenzano A, Sozzi E, Birtele M, Kajtez J, Giacomoni J, Nilsson F, et al. Single-cell transcriptomics captures features of human midbrain development and dopamine neuron diversity in brain organoids. Nat Commun. 2021;12(1):7302. 34911939 10.1038/s41467-021-27464-5 PMC8674361

[53] Park DS, Kozaki T, Tiwari SK, Moreira M, Khalilnezhad A, Torta F, et al. iPS-cell-derived microglia promote brain organoid maturation via cholesterol transfer. Nature. 2023;623(7986):397–405. 37914940 10.1038/s41586-023-06713-1

[54] Mansour AA, Goncalves JT, Bloyd CW, Li H, Fernandes S, Quang D, et al. An in vivo model of functional and vascularized human brain organoids. Nat Biotechnol. 2018;36(5):432–41. 29658944 10.1038/nbt.4127 PMC6331203

[55] Sun XY, Ju XC, Li Y, Zeng PM, Wu J, Zhou YY, et al. Generation of vascularized brain organoids to study neurovascular interactions. Elife. 2022:11:e76707. 35506651 10.7554/eLife.76707 PMC9246368

[56] Broadfield LA, Pane AA, Talebi A, Swinnen JV, Fendt SM. Lipid metabolism in cancer: new perspectives and emerging mechanisms. Dev Cell. 2021;56(10):1363–93. 33945792 10.1016/j.devcel.2021.04.013

[57] Jin HR, Wang J, Wang ZJ, Xi MJ, Xia BH, Deng K, et al. Lipid metabolic reprogramming in tumor microenvironment: from mechanisms to therapeutics. J Hematol Oncol. 2023;16(1):103. 37700339 10.1186/s13045-023-01498-2 PMC10498649

[58] Peng H, Wang Y, Luo W. Multifaceted role of branched-chain amino acid metabolism in cancer. Oncogene. 2020;39(44):6747–56. 32978521 10.1038/s41388-020-01480-z PMC7606751

[59] Reily C, Stewart TJ, Renfrow MB, Novak J. Glycosylation in health and disease. Nat Rev Nephrol. 2019;15(6):346–66. 30858582 10.1038/s41581-019-0129-4 PMC6590709

[60] Propper DJ, Balkwill FR. Harnessing cytokines and chemokines for cancer therapy. Nat Rev Clin Oncol. 2022;19(4):237–53. 34997230 10.1038/s41571-021-00588-9

[61] Xu S, Wang Q, Ma W. Cytokines and soluble mediators as architects of tumor microenvironment reprogramming in cancer therapy. Cytokine Growth Factor Rev. 2024;76:12–21. 38431507 10.1016/j.cytogfr.2024.02.003

[62] Goldman SA. Stem and progenitor cell-based therapy of the central nervous system: hopes, hype, and wishful thinking. Cell Stem Cell. 2016;18(2):174–88. 26849304 10.1016/j.stem.2016.01.012 PMC5310249

[63] Xue J, Wu Y, Bao Y, Zhao M, Li F, Sun J, et al. Clinical considerations in Parkinson’s disease cell therapy. Ageing Res Rev. 2023;83: 101792. 36402405 10.1016/j.arr.2022.101792

