# Supplementary material for: A tumorigenicity evaluation platform for cell therapies based on brain organoids
Source: Transl Neurodegener. 2024 Oct 29;13:53. doi: 10.1186/s40035-024-00446-5 (PMC11520457; doi:10.1186/s40035-024-00446-5)
Supplement: Supplementary file 1 — Additional file 1: Fig. S1 The long-term cell fate of mDA cells after being injected into the brain organoids. Fig. S2 H&E staining of brain organoids and mouse brain sections after mDA cells injection. Fig. S3 Live images of brain organoids after hPSCs injection. Table S1: Antibodies used in this study [file 40035_2024_446_MOESM1_ESM.docx]

**Additional file 1**

**
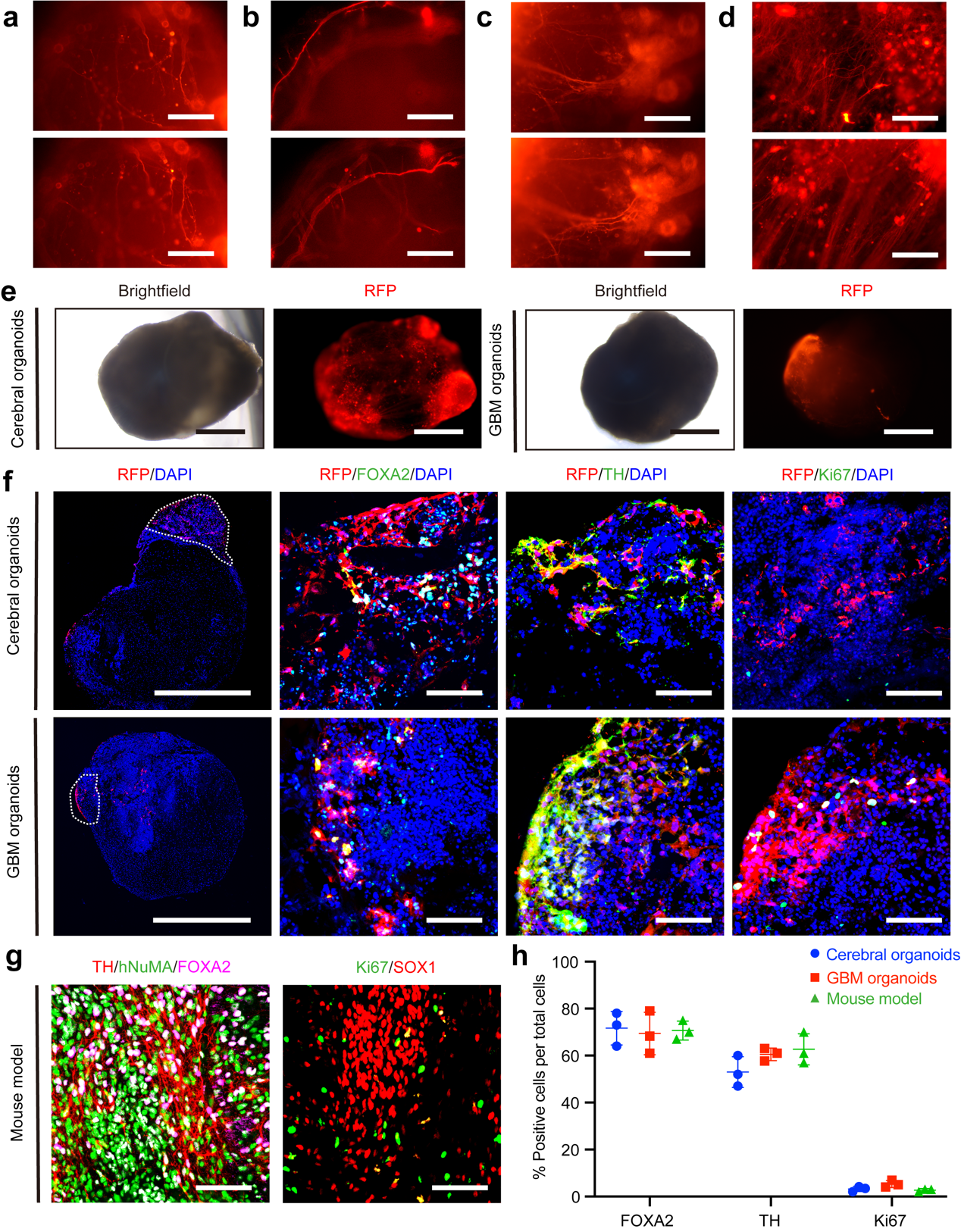
**

**Fig. S1 The long-term cell fate of mDA cells after being injected into the brain organoids.** Additional live images of mDA cells injected into the cerebral organoids (**a, c**) and GBM organoids (**b, d**) at 4 weeks (**a, b**) and 12 weeks (**c, d**) post-injection. Scale bars: 200 μm. **e** The bright-field and live images of mDA cells into the cerebral organoids and GBM organoids post 12-week injection. Scale bars: 1000 μm. **f** The immunofluorescence images of FOXA2, TH, and Ki67 of mDA cells after being injected into the brain organoids. Scale bar: leftmost column 1000 μm, right three columns 80 μm. **g** Immunofluorescence staining of hNuMA, FOXA2, TH, Ki67, and SOX1 in NOD SCID mice at 12 weeks after transplantation of mDA cells. Scale bars: 80 μm. **h.** Quantification of FOXA2^+^, TH^+^, and Ki67^+^ populations in the cerebral organoids, GBM organoids, and mouse model at 12 weeks after injection of mDA cells. Data are presented as mean ± SD, *n* = 3 for each group. Data were analyzed using a two-tailed t-test.

**
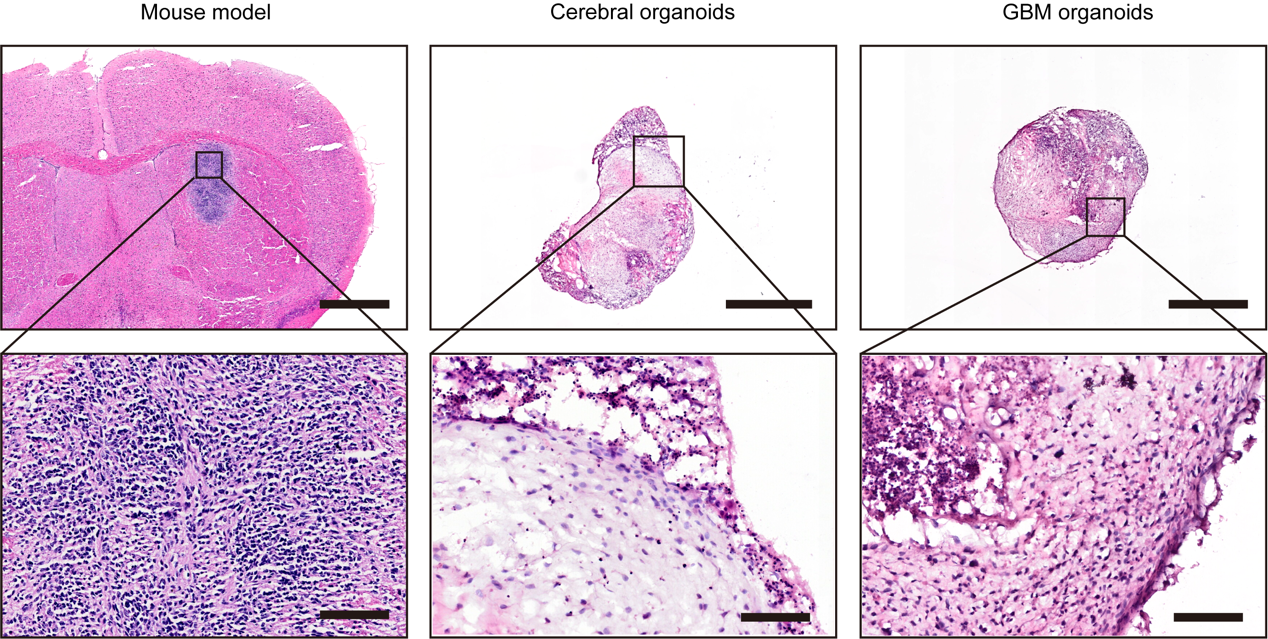
**

**Fig. S2 H&E staining of brain organoids and mouse brain sections after mDA cells injection.** Scale bars, upper 1000 μm, lower 100 μm.

**
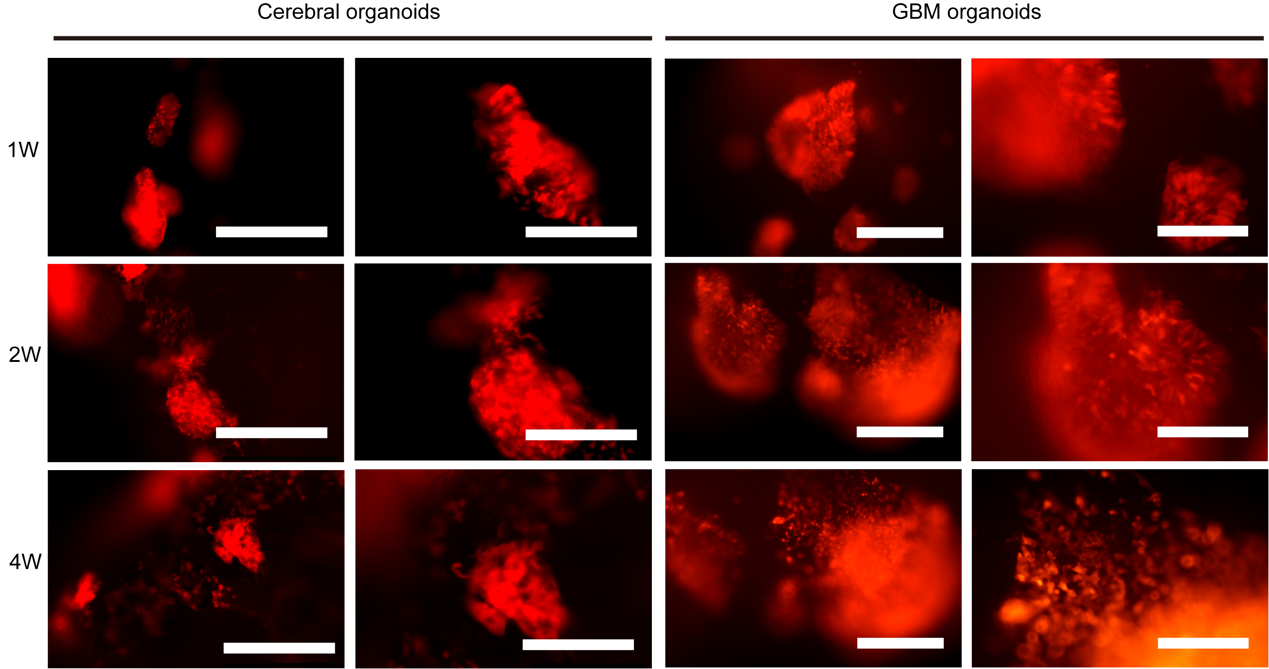
**

**Fig. S3 Live images of brain organoids after hPSCs injection.** Scale bars: 200 μm.

**Table S1: Antibodies used in this study**

| **Antibody** | **Company** | **Cat #** |
| --- | --- | --- |
| FOXA2 | R&D systems | AF2400-SP |
| LMX1A | Abcam | ab139726 |
| EN1 | DSHB | 4G11 |
| TH | Millipore | AB9702 |
| MAP2 | Cell signaling technology | 4542 |
| Tuj-1 | Biolegend | 801201 |
| Nestin | Santa Cruz | sc-23927 |
| Doublecortin | Abcam | ab18723 |
| GFAP | Cell signaling technology | 3670 |
| S100β | Abcam | ab52642 |
| RFP | Rockland | 600-401-379 |
| Luciferase | Novus Biologicals | NB100-1677 |
| OCT4 | Cell signaling technology | 2750 |
| OCT4 | Santa Cruz | sc-5279 |
| Ki67 | Cell signaling technology | 9449 |
| Ki67 | Abcam | ab15580 |
| hNuMA | Abcam | ab97585 |
| SOX1 | R&D systems | AF3369-SP |
| Hoechst 33258 | Abcam | ab228550 |
| Cy™3 Anti-Chicken | Jackson ImmunoResearch | 103-165-155 |
| Alexa Flour 647 Anti-Mouse | Jackson ImmunoResearch | 715-606-151 |
| Alexa Fluor 568 Anti-Rabbit | Invitrogen | A10042 |
| Alexa Fluor 647 Anti-Rabbit | Invitrogen | A31573 |
| Alexa Flour 488 Anti-Rabbit | Jackson ImmunoResearch | 711-545-152 |
| Rhodamine Anti-Goat | Jackson ImmunoResearch | 705-296-147 |
| Goat Anti-Rabbit IgG, HRP conjugated | CWBIO | CW0103S |
| Rabbit Anti-Goat IgG, HRP conjugated | Yeasen | 33701ES60 |
